# Investigation of thrombin concentration at the time of clot formation in simultaneous thrombin and fibrin generation assays

**DOI:** 10.1038/s41598-023-47694-5

**Published:** 2024-04-22

**Authors:** Ivan D. Tarandovskiy, Stepan S. Surov, Leonid A. Parunov, Yideng Liang, Wojciech Jankowski, Zuben E. Sauna, Mikhail V. Ovanesov

**Affiliations:** grid.417587.8 0000 0001 2243 3366 U.S. Food and Drug Administration, 10903 New Hampshire Avenue, Silver Spring, MD 20993 USA

**Keywords:** Biochemistry, Biological techniques, Biophysics, Computational biology and bioinformatics, Systems biology, Cardiovascular biology, Predictive markers

## Abstract

Thrombin generation (TG) and fibrin clot formation represent the central process of blood coagulation. Up to 95% of thrombin is considered to be generated after the clot is formed. However, this was not investigated in depth. In this study, we conducted a quantitative analysis of the Thrombin at Clot Time (TCT) parameter in 5758 simultaneously recorded TG and clot formation assays using frozen plasma samples from commercial sources under various conditions of activation. These samples were supplemented with clotting factor concentrates, procoagulant lipid vesicles and a fluorogenic substrate and triggered with tissue factor (TF). We found that TCT is often close to a 10% of thrombin peak height (TPH) yet it can be larger or smaller depending on whether the sample has low or high TPH value. In general, the samples with high TPH are associated with elevated TCT. TCT appeared more sensitive to some procoagulant phenotypes than other commonly used parameters such as clotting time, TPH or Thrombin Production Rate (TPR). In a minority of cases, TCT were not predicted from TG parameters. For example, elevated TCT (above 15% of TPH) was associated with either very low or very high TPR values. We conclude that clotting and TG assays may provide complementary information about the plasma sample, and that the TCT parameter may serve as an additional marker for the procoagulant potential in plasma sample.

## Introduction

Blood coagulation results in clot formation which prevents blood loss from damaged vessels and has other functions in vessel development and repair. The currently available functional hemostasis assays focus on detection of ether fibrin clot formation or generation of thrombin activity.

Assays based on clot formation include the commonly used tests of plasma clotting time, e.g., Activated Partial Thromboplastin Time (aPTT) and Prothrombin Time (PT)^1^ as well as point-of care methods that measure blood clot elasticity^2,3^. The kinetics of clot formation in plasma can also be extracted from the clot turbidity (light absorbance) data in an aPTT- and PT-based Clot Waveform analysis method or microplate-based clot formation and fibrinolysis assays. However, these Fibrin Generation (FG) clot-based assays capture only a portion of the events that take place during blood coagulation because clot formation is rapid and short-lived compared to the substantially longer process of thrombin generation^4^.

Assaying the kinetics of thrombin generation (TG) during clotting of blood plasma is widely used for clinical and scientific research purposes^5,6^. The most frequently used approach to measure TG is to measure fluorescence from 7-amino-4-methylcoumarine (AMC) following cleavage of an synthetic thrombin specific substrate by generated thrombin^7,8^. Investigation of thrombin is diagnostically relevant since thrombin provides the enzymatic reaction of clot formation, plays a central role in the positive and negative feedback loops of the coagulation cascade [4] and participates in many other related functions, including regulation of fibrinolytic system, activation of platelets, stimulation of repair mechanisms on endothelial cells and inflammation responses.

In commonly used clot-based and TG-based methods, two main processes of blood coagulation – clot formation and TG – are investigated independently. Although occasional reports aimed to measure clot formation and TG simultaneously^9–14^, there is no a wide-spread assay in which TG and Fibrin Generation (FG) are measured simultaneously. Moreover, the TG/FG assays in the aforementioned reports analyzed TG and FG parameters separately. A single parameter that would incorporate both of FG and TG has not yet been published and investigated. Though TG and FG frequently do not correlate with each other, they are biochemically closely related. A parameter that reflects TG and FG relationship could provide more information about the regulation of blood coagulation as it would be sensitive to both TG and FG.

In this study, we examine a parameter that incorporates both TG and FG, namely, the concentration of thrombin measured at the time of clot formation or, in other words, Thrombin at Clot Time (TCT). Besides providing information on TG and FG, the parameter TCT has additional utility. For example, TCT may indicate concentration of thrombin for which diffusion is not limited by adsorbtion to clot, compared to diffusion of thrombin that is generated after the clot is formed. The hypothesis that thrombin generated prior to fibrin clot formation diffuses more rapidly than thrombin generated inside a clot is supported by previous studies ﻿[15, 16, 17, 18, 19]. Coagulation proteases inside the fibrin clot were shown to diffuse slower than ones in the unclotted plasma^15^. Similarly, adsorption of thrombin to fibrin reduces thrombin generation by plasma^16–19^. Thus, assessing TCT possibly allows estimating thrombin amount that can propagate through the blood vessel. The propagation of the clot is investigated directly by spatial-resolved assay systems (e.g., thrombodynamics), but they are not widely used because of their complexity^20,21^. Furthermore, fibrin clot structure was also shown to depend on the concentration of thrombin that cleaves fibrinogen^22^. Higher thrombin concentrations produce relatively less turbid and less permeable fibrin clots while the clot produced by lower concentrations of thrombin is more permeable and less stable^23–25^. Thus, measuring TCT in a simultaneous TG/FG assay may be useful for indirect assessment of the stability and permeability of fibrin clots in tested samples.

Among the reasons TCT was missed out (aside from the lack of usage of simultaneous TG/FG measurement systems), is the prevailing belief that rapid TG phase starts after fibrin clot formation. Indeed, pioneering studies of Mann et al.^26^ showed that up to 90% of all generated thrombin is formed inside the clot. This has led to an assumption that only a small fraction of thrombin is generated prior to clot formation. However, Mann’s whole blood studies^26^ did not use the conditions^8,27^ that are conventional in TG assays. Furthermore, the influence of coagulation factors and inhibitors on thrombin concentration at clot time was not investigated. In the present study, we have analyzed 5758 simultaneous TG/FG measurements carried out in our laboratory under a wide range of triggering conditions and in the presence of various coagulation agents at different concentrations. In these experiments, we calculated absolute TCT values in nanomoles (aTCT) and the ratio between TCT and Thrombin Peak Height in percent (rTCT, TPH correspondingly). We evaluated the dependence of TCT on various experimental conditions in comparison with commonly used TG parameters such as TPH and Thrombin Production Rate (TPR). Taken together our findings indicate that TCT can be a useful additional parameter for the identification of abnormalities in blood coagulation.

## Results

### Representative TG and FG curves with different TCT values

**Figure 1:**
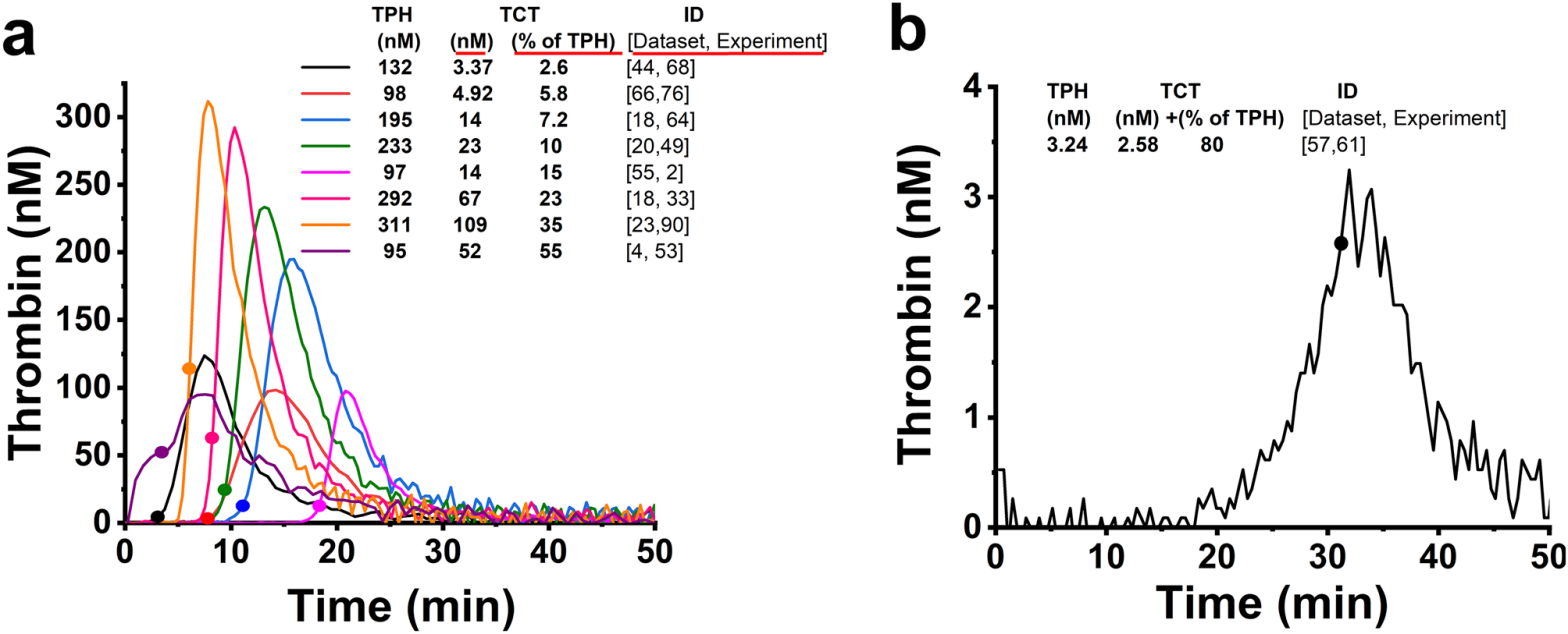
(**a**, **b**) Representative TG obtained from several experiments used in the study. Circles on the curves (**a**, **b**) indicate a concentration of thrombin at the clot times (TCT values). Each TG curve with an accompanied FG curve can be found in Fig. S2. The notation of [Dataset and experiment] indicates the reference to the experiment for the shown curves. The conditions of experiments can be found in Table S3.

TG measurements from 5758 samples were analyzed. Most of these experiments were focused on: (i) effects of wild type and bioengineered variants of recombinant Factor VIIa (rFVIIa) in Factor VIII-deficient plasma^28^, (ii) effects of addition of normal plasma to FVIII deficient plasma, (iii) effects of Factor IX (FIX) and IXa (FIXa) on FIX-deficient and normal plasma^12^, (iv) effects of platelet-derived microvesicles (PMV) on normal plasma, (v) effects of activated Factor XI (FXIa) contamination in immune globulin products on FXI-deficient plasma^11,29^, and (vi) effect of calcium chloride on TG in normal plasma^30^ (Fig. S1). Additional studies with fewer samples that were used in our analyses are shown in Table S1. All experimental conditions and TG parameters values can be found in the Table S3. Figure 1 exemplifies the variety of the conditions and results obtained. In some samples 90% of thrombin was indeed generated after clot time (TCT values about 10% of TPH, Fig. 1a, Fig. S2a–d), as could be expected from previous observations^26^. However, in some experiments, the TCT values were 15% of TPH or higher (Fig. 1a, Fig. S2e–i), indicating that a considerable fraction of TG may occurs prior to the formation of fibrin clot. Our observations show, that elevated thrombin generation prior to clot formation can occur when the TPH value is high (Fig. 1a, Fig. S2e–h) or extremely low (Fig. 1b, Fig. S2i). The conditions of each experiment can be found in Table S2.

### Under certain conditions, thrombin generation during clot formation reaches comparatively high values

**Figure 2:**
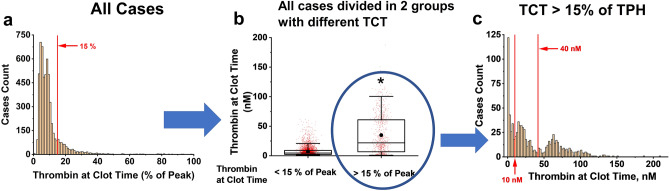
(**a**) The histogram of TCT (% of TPH) distribution for all 5758 experiments analyzed in the study. (**b**) Box plot of aTCT values in the cases of TCT below or higher 15% of TPH. The lowest single horizontal whisker line, the lower box border line, the higher box border line and the highest single whisker line represent the 5th, 25th, 75th and 95th percentiles, respectively. The horizontal line inside the box represents the median. (•) represents the mean value, (*) represents the significant difference obtained by the Mann–Whitney test with P set as 0.05. (**c**) The histogram of the distribution of aTCT values in the cases when TCT is 15% of TPH and higher.

A distribution of TCT values for all 5758 analyzed samples is depicted in Fig. 2a. The median TCT value was observed at ~ 5% of the TG peak. A majority of samples had TCTs below 10% of the peak which is consistent with the previous study^26^ that showed 90% of TG occurs after clot formation. Nonetheless a tail of the distribution was formed by a group of samples with TCT values > 15% of TPH (Fig. 2a). The value 15% was chosen as a cut-off for a Control group (4898 samples with TCT < 15%, 85.1% of total). We compared the Control group and and the group of samples that formed the tail and found that the samples with TCT >15% demonstrate greater diversity (Figure 2b). In Fig. 2c, we identify 3 peaks in the group with a relative TCT of > 15%, stratified on the basis of the absolute TCT value (aTCT): aTCTs with values < 10 nM (LowTCT group), aTCTs with values between 10 and 40 nM (MedTCT group), and aTCTs with values > 40 nM (HighTCT group). A change in TCT cut-off value for control group qualitatively did not change TCT distribution in the subpopulation of TCT values elevating the border (Fig. S3). Table 1 shows the number of samples in each of these three groups and compared to the Control group.

**Table 1:** Total count of cases in High TCT groups and the Control Group.

Group		LowTCT	MedTCT	HighTCT	Control
Thrombin at Clot Time	% of TPH	≥ 15			< 15
	Absolute values, nM	< 10	10–40	≥ 40	All values
Total Count		257	295	308	4898
% of total		4.5	5.1	5.3	85.1

### Potential association between TCT values and hyper- and hypo- coagulation states

**Figure 3:**
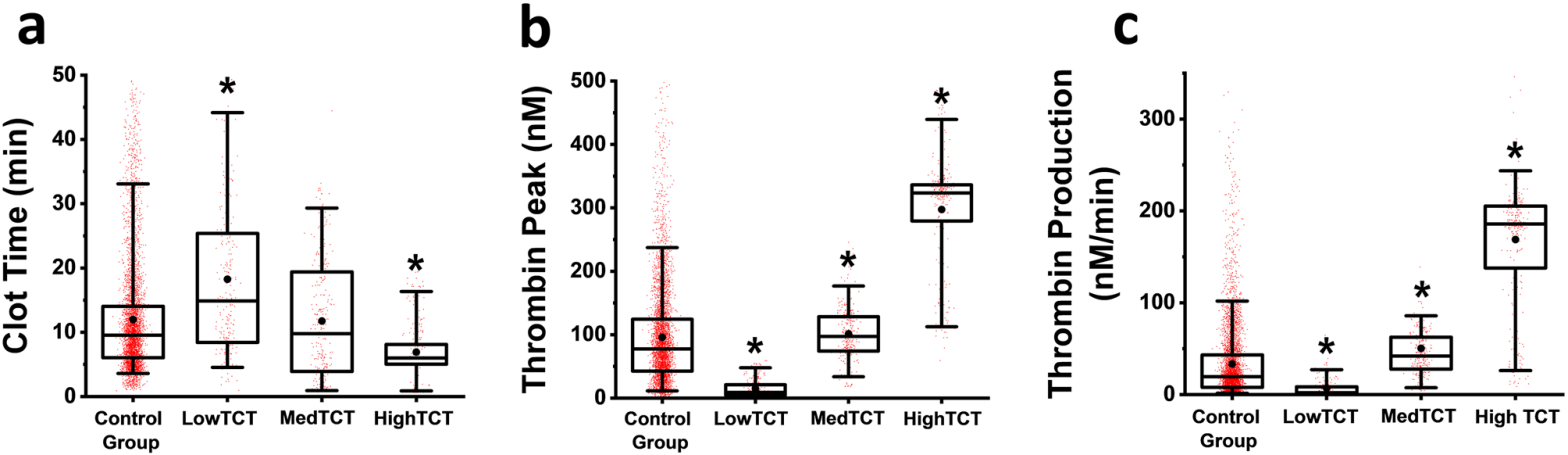
Box plots of Clot Time (**a**), TPH (**b**) and TPR (**c**) values in the control group (rTCT < 15% of TPH), and the group of samples with rTCT > 15% of TPH, subdivided into 3 subgroups according to their aTCT values: LowTCT (aTCT < 10 nM), ﻿MedTCT (aTCT between 10 and 40 nM), HighTCT (aTCT > 40 nM). On the box plot, the lower whisker, the lower box border line, the higher box border line and the higher whisker represent the 5th, 25th, 75th and 95th percentiles, respectively. The horizonal line inside the box denotes the median. (•) represents the mean value, and (*) indicates a significant difference from the Control Group. Statistics was obtained by the Mann–Whitney test with P set as 0.05 with Bonferroni correction for 4 samples.

Figure 3 demonstrates that the LowTCT (< 10 nM) group has significantly higher clot times (Fig. 3a), significantly lower TPHs (Fig. 3b) and significantly lower TPRs (Fig. 3c) compared to the Control group. Collectively, these data indicate reduced coagulation activity in the plasma samples from the LowTCT group. Both MedTCT and HighTCT groups have significantly higher TPH (Fig. 3b) and TPR (Fig. 3c) compared to the Control group suggesting hypercoagulation.

### Correlations between TPH and TCT values for the Control, LowTCT, MedTCT and HighTCT groups

**Figure 4:**
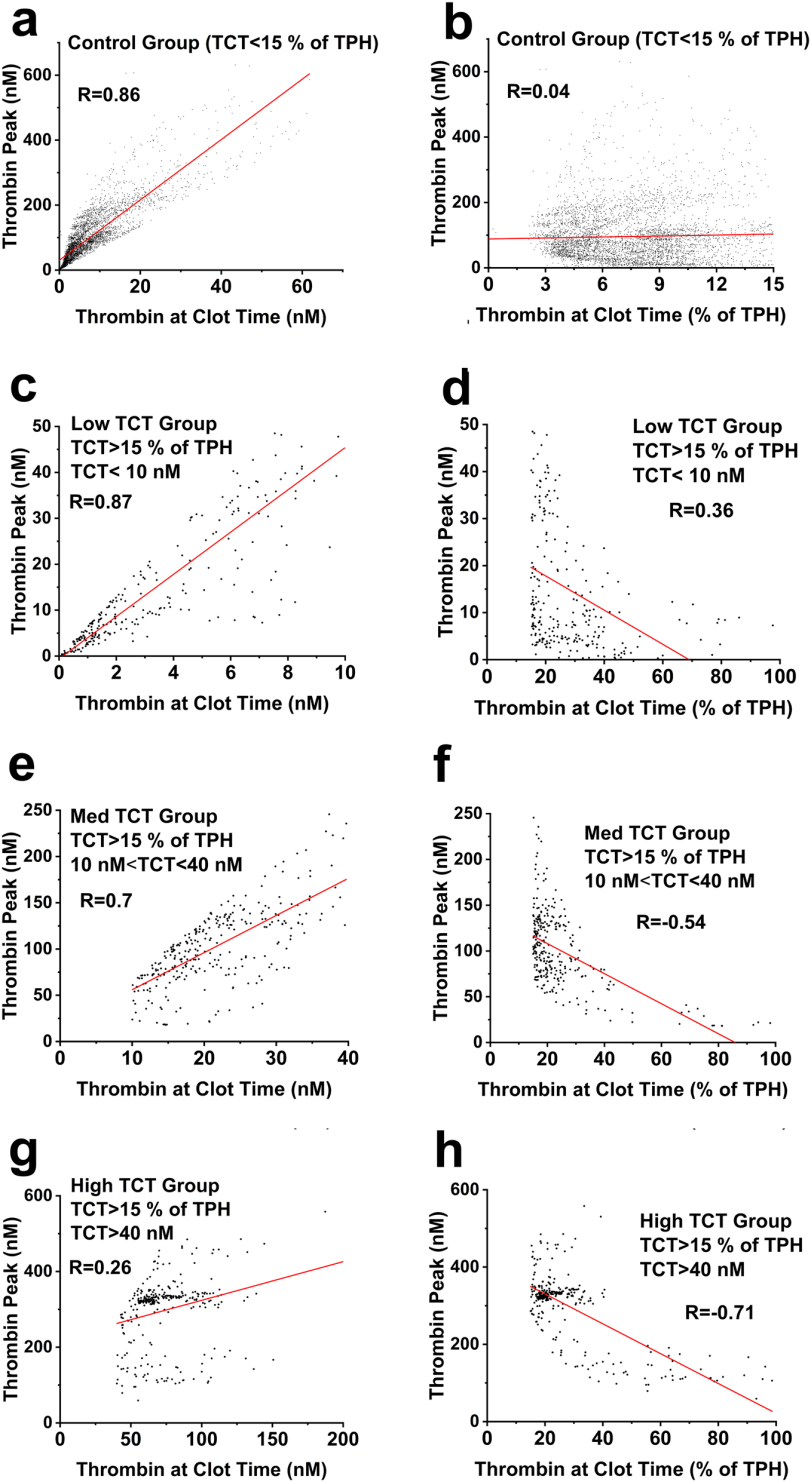
Correlations between TCT and TPH values in Control (**a**, **b**), LowTCT (**c**, **d**), MedTCT (**e**, **f**) and HighTCT (**g**, **h**) groups.

We found that aTCT correlated well with TPH for the control, LowTCT, and MedTCT groups, but not for the HighTCT group (Fig. 4a,c,e,g). These results indicate that aTCT values can be predictive of TPH when certain conditions are met.. Importantly, a correlation between rTCT and TPH was not observed (Fig. 4b,d,f,h).

### TCT value depends on the presence of procoagulant agents

This study includes analysis of samples supplemented with procoagulant (FVIIa, FIX, FIXa, FXIa, TF) and/or anticoagulant agents at various concentrations. To investigate how TG parameters reflect the potential procoagulant activity caused by addition of multiple agents, we established an in-house procoagulant score (PS) for each experiment. PS was designed as a sum of so called “procoagulant and anticoagulant markers” that corresponded to added pro- and anticoagulant agents and their concentrations (Table 2). Each procoagulant marker adds 1 to PS. Conversely, each anticoagulant marker subtracts 1 from the PS. These markers were set with respect to known normal concentrations of procoagulant and anticoagulant agents used in the study. A plasma with a factor deficiency was considered having an anticoagulant markers as opposed to plasmas from healthy donors (procoagulant markers). The agents with weak effect on TG (Corn Trypsin Inhibitor, Carboxypeptidase Inhibitor, Fibrinogen) were excluded from PS. To illustrate the PS calculation, consider the experiment performed  in FVIII-deficient plasma supplemented with 200 nM FVIIa, 0.5 pM TF and 4 μM of phospholipids. The PS is calculated by summing individual contributions: − 1 (FVIII-deficient plasma) + 1 (FVIIa addition) + 1 (FVIIa ≥ 200 nM) resulting in a PS of 1. Following PS calculation, we correlated PS with the observed TPH, TPR and aTCT values.

**Table 2:** Procoagulant and anticoagulant markers used in the design of procoagulant score.

**Procoagulant Markers (each one adds 1 to PS)**
Normal Plasma addition to deficient plasma
FVIIa addition
FVIIa ≥ 200 nM
FVIIa Mutant ≥ 200 nM
TF ≥ 1 pM
TF ≥ 5 pM
FXIa ≥ 1 pM addition
FIX ≥ 0.1 IU/ml addition
FIX ≥ 0.25 IU/ml addition
FIXa ≥ 0.0016 IU/ml addition
Immune Globulins addition
Platelet Microvesicles addition
TG Phospholipids > 4 μM
Prothrombin Complex Concentrate > 0.25 IU/ml
**Anticoagulant Markers (each one subtracts 1 from PS)**
FVIII or FIX Deficient Plasma
No TF
TG Phospholipids < 4 μM
Non-Standard Calcium concentration (out of the range 10–12 mM)
Antithrombin ≥ 0.5 IU/ml addition
Apixaban

**Figure 5:**
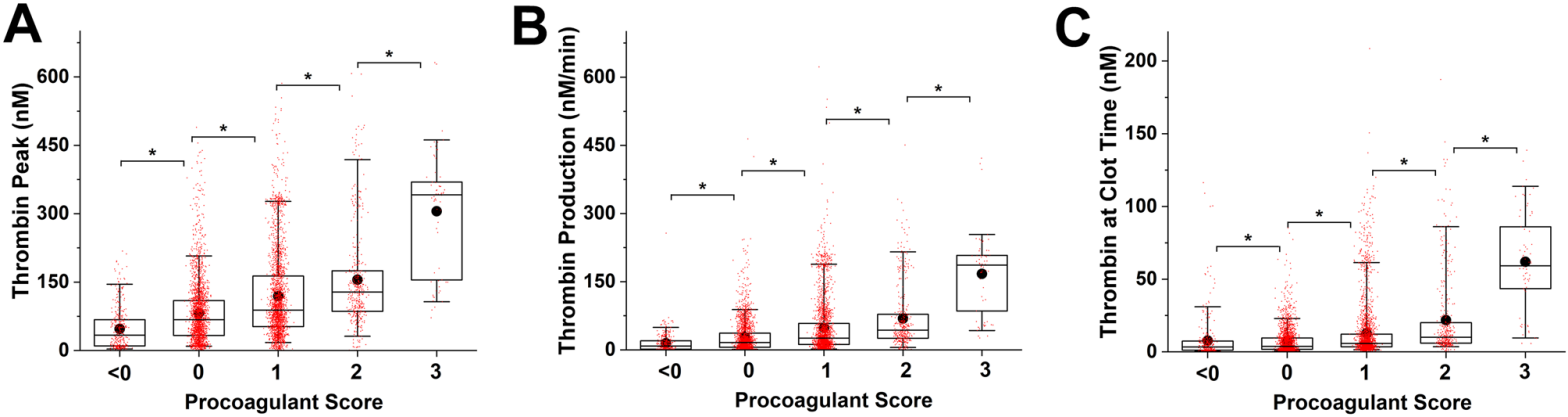
The box plots of TPH (**a**), TPR (**b**) and aTCT (**c**) values with different PS. The lower whisker, the lower box border line, the higher box border line and the higher whisker represent 5th, 25th, 75th and 95th percentiles respectively. The line inside the box denotes the median. (•) represents the mean value, (*) indicates significant difference. Statistics was obtained by the Mann–Whitney test with P set as 0.05 with Bonferroni correction for 6 samples.

Figure 5 shows correlations of PS and TG parameters (TP, TPR and aTCT) for all experiments included in the study. All three parameters differ in the PS range from below 0 to 1 and when PS reaches 3. However, the mean and median values from different TG parameters tend to reflect PS differently (Table S2). TPR and aTCT were more sensitive to change in PS.

### Examples of interplay between TCT, TPH and TPR. FIX titration in FIX deficient plasma

**Figure 6:**
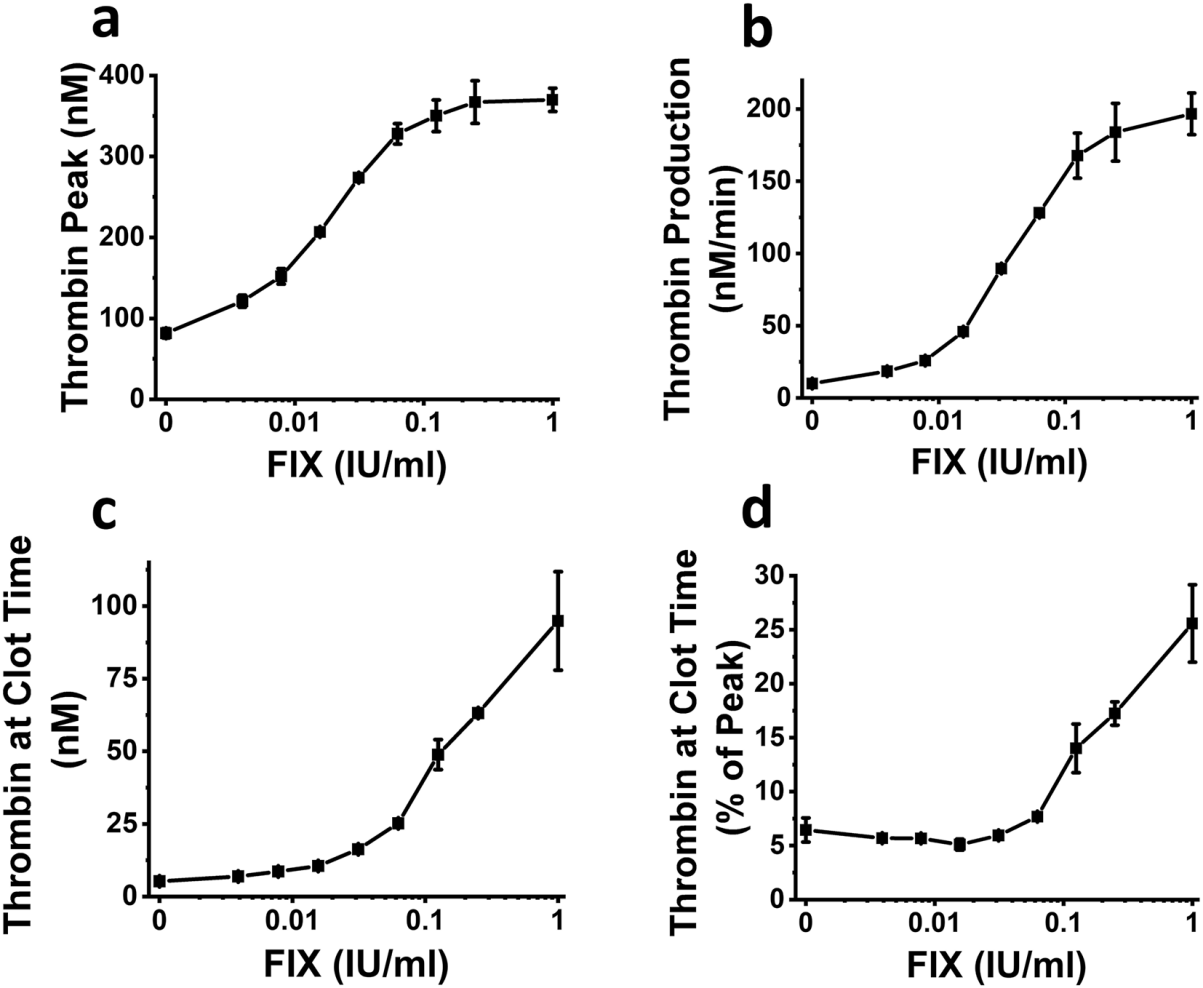
The dependencies of TPH (**a**), TPR (**b**) and TCT (**c**–**d**) on FIX concentration in FIX-deficient plasma. Experiments 1–15 from dataset 70 in Table S3. Each dot represents a mean value of two duplicate experiments with SD values.

To evaluate utility of TPH, TRP, aTCT, and rTCT we analyzed sensitivity of these parameters to procoagulant stimuli. Figure 6 demonstrates the example of responses of TPH (Fig. 6a), TPR (Fig. 6b) and TCT (Fig. 6c–d) to increasing concentrations of FIX added to FIX-deficient plasma. At the FIX concentrations below 0.01 IU/ml (severe hemophilia B^31,32^), TPH and TPR demonstrate higher sensitivity to FIX than aTCT and rTCT. In contrast, at FIX concentrations above 0.01 IU/mL (which corresponds to moderate and mild hemophilia B^32^), TCT demonstrates better sensitivity to FIX concentrations.

### Examples of interplay between TCT, TPH and TPR. TG phospholipid vesicles titration in normal plasma

Figure S4 shows the dependencies of TPH (Fig. S4a), TPR (Fig. S4b), aTCT and rTCT (Fig. S4c-d) on the concentration of commercially available phospholipids that are used in TG assay to boost thrombin formation. In line with Fig. 6, in the case of titration of phospholipids, aTCT and rTCT demonstrates high sensitivity to the higher concentrations of an added agent.

### Examples of interplay between TCT, TPH and TPR. Two types of FVIIa titration in FVIII deficient plasma

Figure S5 demonstrates a response of TG assay in FVIII deficient plasma to increasing concentrations of human recombinant FVIIa^33^ and its bioengineered analog (NN1731)^34^. In this case, TPH and TPR were sensitive to both types of FVIIa in the wide range of concentrations (Fig. S5a–b). However, they do not show a large difference between each type. In contrast, aTCT and rTCT were sensitive to 100–1000nM NN1731, but not to recombinant wild type FVIIa. 

## Discussion

In the present study, we evaluated 5,758 simultaneously recorded TG and clot formation assays carried out under various conditions. In the past, it was believed that only negligible amounts of thrombin are generated at (or prior to) the time of clot formation^22,26^. In approximately 15% of the evaluated assays, thrombin levels generated prior to clot formation can reach substantial values compared to TPH. Importantly, thrombin generation prior to clot formation is associated with abnormal conditions of hypo- and hypercoagulability. Our results suggest that TCT could be a valuable parameter to study various coagulopathies.

We hypothesize, that abnormal TCTs in samples with decreased or elevated procoagulant activity may be a result of imbalance in thrombin and fibrin production rates. Specifically, in cases with reduced procoagulant or increased anticoagulant activity in plasma, lower rates of thrombin generation may have a significantly greater impact on reducing TPH than TCT, because as few as several nM of thrombin are sufficient to form a fibrin clot^22^. Conversely, if thrombin is generated rapidly, but the rate of clot formation is limited by the concentration of fibrinogen and fibrin polymerization, the concentration of thrombin generated before the clot is formed, can reach high aTCT and rTCT.

In this study we demonstrated that the amount of thrombin formed at clot time (TCT) can be used as an additional parameter of TG to study mechanisms of action of various coagulation agents used in clinics and research. In development of our in-house PS, we attempted to evaluate potential complex effects of different conditions on TG/FG assays. However, the results of some experiments did not match predictions of PS. In many cases experiments with the same PS differed a lot in at least one of parameters (Fig.S4c–d). This can be explained by the fact that many agents that have similar mechanism of action and were assigned the same procoagulant score, in practice, resulted in different TG responses, like it was with two types of FVIIa in Fig. S5. But finally, taken together to the groups with same PS, TG parameters significantly differed between these groups indicating correlation of our in-house designed PS with procoagulant activity (Fig. 5).

Our study included samples where sensitivities of different parameters of TG/FG assay to one agent (e.g., FXIa, FVIIa, FIX) depend on its type and concentration (Figs. 6, S4, S5, more examples can be found in Table S3). The agents that were used in the study act in different coagulation pathways, and therefore can affect TCT, TPH, and TPR in different ways. For instance, when TCT shows higher sensitivity than TPH or TPR, we show that TCT provides an additional tool to evaluate the action of coagulation agents with various mechanisms of action.

In the present study we calculated clot time as the time when optical density from fibrin clot reaches 45% level of its maximal value. Our method to determine CT is consistent with the approach used in some previously published studies^9,10^. The approach to determining clot time, however, varies across instruments, assays, and investigators. For example, clot time can be determined as the time when the optical density reaches its maximum absorbance^35, or determined as the point when the second derivative of the absorbance curve reaches it’s maximum, as it is done in ACL TOP analyzers^. The method used to calculate CT could affect the absolute values of TCT but does not qualitatively change the relation of high TCT with high procoagulant activity of plasma (Fig. S6).

The use of TCT parameter has some limitations, that arise from the requirement to measure fluorescence and absorbance simultaneously. A condition that affect fibrin formation potentially may alter clot time, and, as a result TCT. For example, the use of Tissue Plasminogen Activator (tPA) is common in assays that also measure generation of plasmin aiming to evaluate fibrinolysis^13,14,36–40^. Addition of high concentrations of tPA can affect fibrin concentration and, as a result, light absorption, which can strongly affect the calculation of clot time. The interference of fibrinolysis with clot formation can be minimized by reducing tPA concentration in the reaction mixture. The second limitation arises from the low reading speed (2 to 4 times per minute) of typical plate readers which are used to record the fast transition between the liquid phase (plasma) and the clot. The low time resolution of clot formation results in only a few measurements per transition, and may result in high variability and low reproducibility of CT and of TCT. Development of high-speed readers would overcome this issue.

In our study, we, for the first time, investigated the amount of thrombin that can be generated by the time of clot formation under various conditions. We showed that under lack or excess of coagulation activity, TCT can reach significant values compared to the peak of thrombin. TCT is a promising parameter that can be used in the future studies aiming to investigate abnormal coagulation activity as well as to evaluate effects of various coagulation agents.

## Materials and methods

### Study design

**Figure 7:**
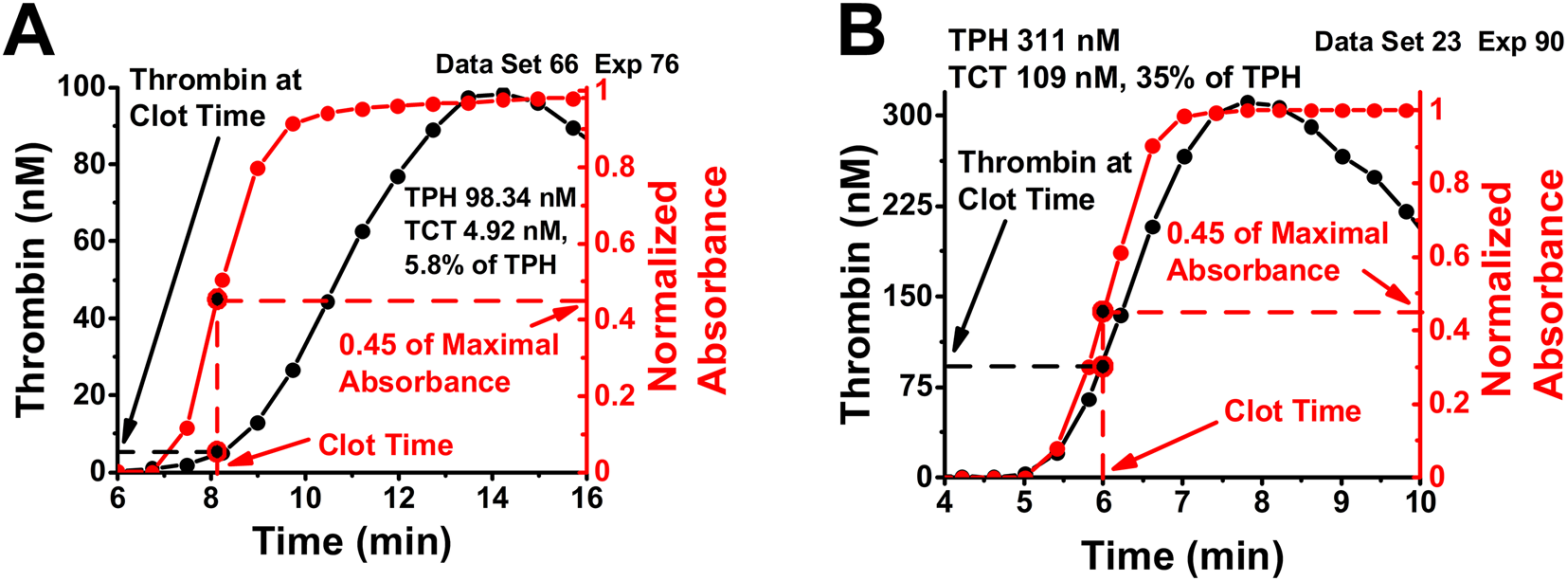
Representative TG and fibrin formation curves with definitions of parameters’ values.

TG/FG assay was performed as previously described^10^. Briefly, blood plasma (50% vol/vol in the final reaction) was substituted with 800 μM of thrombin specific fluorogenic substrate Z-Gly-Gly-Arg-AMC (Bachem Americas, King of Prussia, PA, USA) and Tris-BSA buffer (pH 7.4, Aniara, West Chester, Ohio, USA). Coagulation trigger for each experiment was added as in Table S3. Clotting was initiated by CaCl2 (20.0%, 10 mM final concentration for the most of experiments). Fluoresence (380 nm excitation, 430 nm emission) and absorbance were recorded at 380 nm excitation, 430 nm emission, and 492 nm correspondingly. The Clot Time was calculated as the time when optical density from fibrin clot reaches 45% of its maximal value (Fig. 7). All the agents used in all 5758 experiments analyzed in this study are listed in Table S3 and that sources of them are available upon request. For all calculations MS Excel (Microsoft, Redmond, WA, USA) and Origin (Origin Lab, Northampton, MA, USA) software were used. To obtain statistics, we used Mann–Whitney U-test with P set as 0.05 with Bonferroni correction. In the present study, only commercially available human plasmas were used, and no humans were directly involved.

### Supplementary Information

Supplementary Information 1.

Supplementary Information 2.

## Data Availability

The original datasets that were used in the study are available in Table S3 and from the corresponding author.
