# Supplementary material for: Investigation of thrombin concentration at the time of clot formation in simultaneous thrombin and fibrin generation assays
Source: Sci Rep. 2024 Apr 22;14:9225. doi: 10.1038/s41598-023-47694-5 (PMC11035586; doi:10.1038/s41598-023-47694-5)
Supplement: Supplementary file 1 — Supplementary Information 1. [file 41598_2023_47694_MOESM1_ESM.docx]

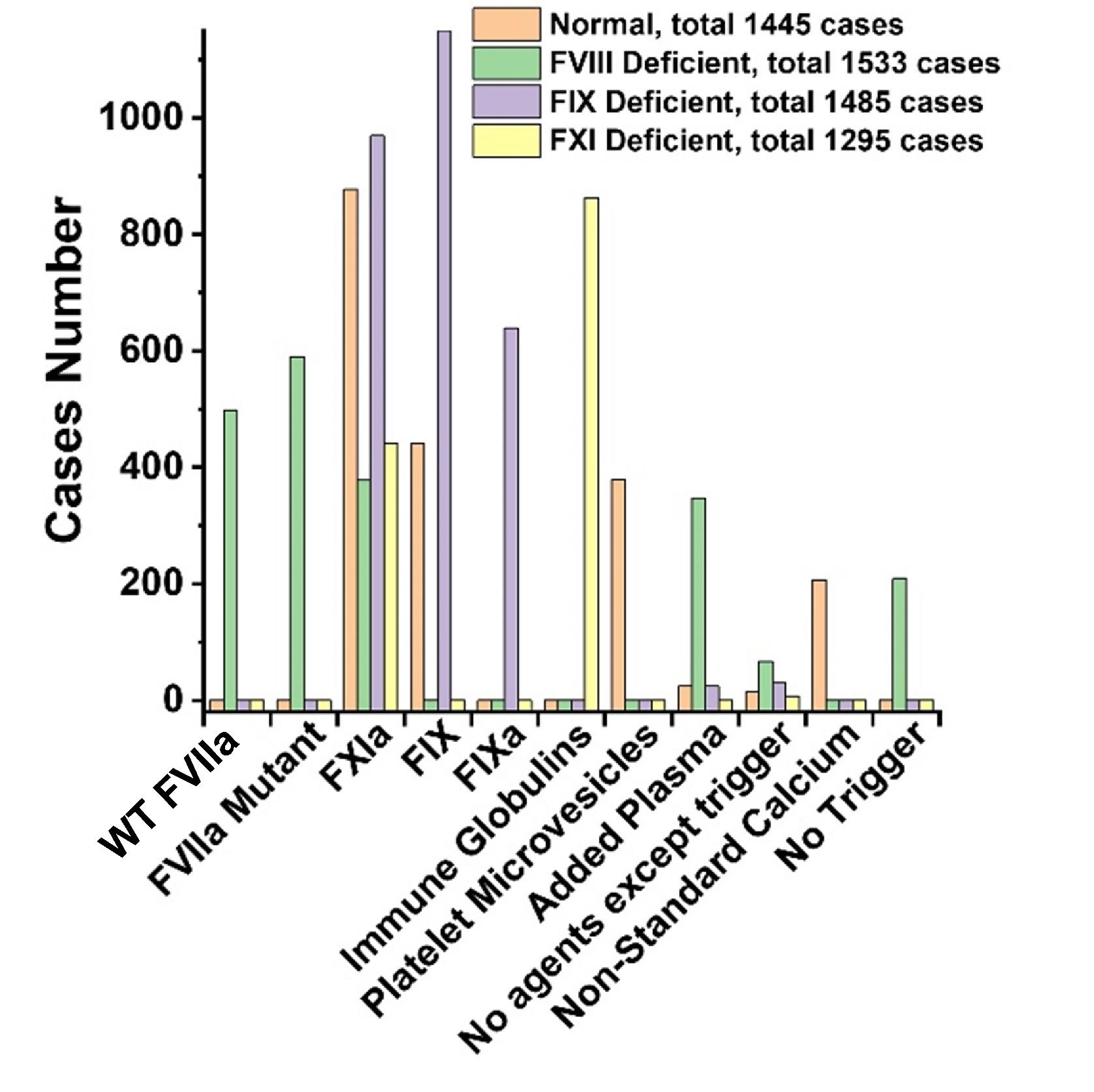


Figure S1. The amount of experiments with coagulation agents that were added to normal, FVIII-, FIX-, and FXI- deficient plasmas. The graph contains the values that exceed 10% of total amount in any type of plasma. All the coagulation agents and triggers are shown in Table 1.

Table S1. Procoagulant and anticoagulant agents and types of coagulation triggers used in the study

| **Plasma** | | **Normal** | | **FVIII Deficient** | | **FIX Deficient** | | **FXI Deficient** | |
| --- | --- | --- | --- | --- | --- | --- | --- | --- | --- |
|  | | **Number of Cases** | **% of total in normal plasmas** | **Number of Cases** | **% of total in FVIIId plasmas** | **Number of Cases** | **% of total in FIXd plasmas** | **Number of Cases** | **% of total in FXId plasmas** |
| **Agents Added to Plasma** | **FVIIa Mutant** | 0 | 0.00 | 590 | 38.49 | 0 | 0.00 | 0 | 0.00 |
|  | **FVIIa wild type** | 0 | 0.00 | 498 | 32.49 | 0 | 0.00 | 0 | 0.00 |
|  | **FXIa** | 877 | 60.69 | 378 | 24.66 | 969 | 65.25 | 442 | 34.13 |
|  | **FIX** | 442 | 30.59 | 0 | 0.00 | 1150 | 77.44 | 0 | 0.00 |
|  | **FIXa** | 0 | 0.00 | 0 | 0.00 | 639 | 43.03 | 0 | 0.00 |
|  | **Immune Globulins** | 0 | 0.00 | 0 | 0.00 | 0 | 0.00 | 862 | 66.56 |
|  | **Platelet Microvesicles** | 379 | 26.23 | 0 | 0.00 | 0 | 0.00 | 0 | 0.00 |
|  | **Added Normal Plasma** | 24 | 1.66 | 346 | 22.57 | 24 | 1.62 | 0 | 0.00 |
|  | **Corn Trypsin Inhibitor** | 894 | 61.87 | 378 | 24.66 | 44 | 2.96 | 0 | 0.00 |
|  | **Carboxypeptidase Inhibitor** | 81 | 5.61 | 0 | 0.00 | 0 | 0.00 | 0 | 0.00 |
|  | **Thrombomodulin** | 49 | 3.39 | 0 | 0.00 | 42 | 2.83 | 0 | 0.00 |
|  | **Antithrombin III** | 10 | 0.69 | 0 | 0.00 | 0 | 0.00 | 0 | 0.00 |
|  | **Apixaban** | 0 | 0.00 | 48 | 3.13 | 0 | 0.00 | 0 | 0.00 |
|  | **Fibrinogen** | 13 | 0.90 | 0 | 0.00 | 0 | 0.00 | 0 | 0.00 |
|  | **Multiple Factors&Inhibitors Concentrates** | 100 | 6.92 | 0 | 0.00 | 0 | 0.00 | 0 | 0.00 |
| **Non-Standard Plasma Dilutions** | | 68 | 4.71 | 0 | 0.00 | 0 | 0.00 | 0 | 0.00 |
| **No additions except trigger** | | 14 | 0.97 | 67 | 4.37 | 30 | 2.02 | 7 | 0.54 |
| **Coagulation Trigger** | **Recombiplastin** | 1445 | 100.00 | 1056 | 68.88 | 1485 | 100.00 | 1295 | 100.00 |
|  | **Soluble Tissue Factor** | 0 | 0.00 | 245 | 15.98 | 0 | 0.00 | 0 | 0.00 |
|  | **Non-standard CaCl_2_ Concentration** | 206 | 14.00 | 0 | 0.00 | 0 | 0.00 | 0 | 0.00 |
|  | **No TG lipids added** | 32 | 2.21 | 67 | 4.37 | 0 | 0.00 | 0 | 0.00 |
|  | **No trigger** | 0 | 0.00 | 208 | 13.57 | 0 | 0.00 | 0 | 0.00 |
| **Total Cases Count** | | **1445** | | **1533** | | **1485** | | **1295** | |


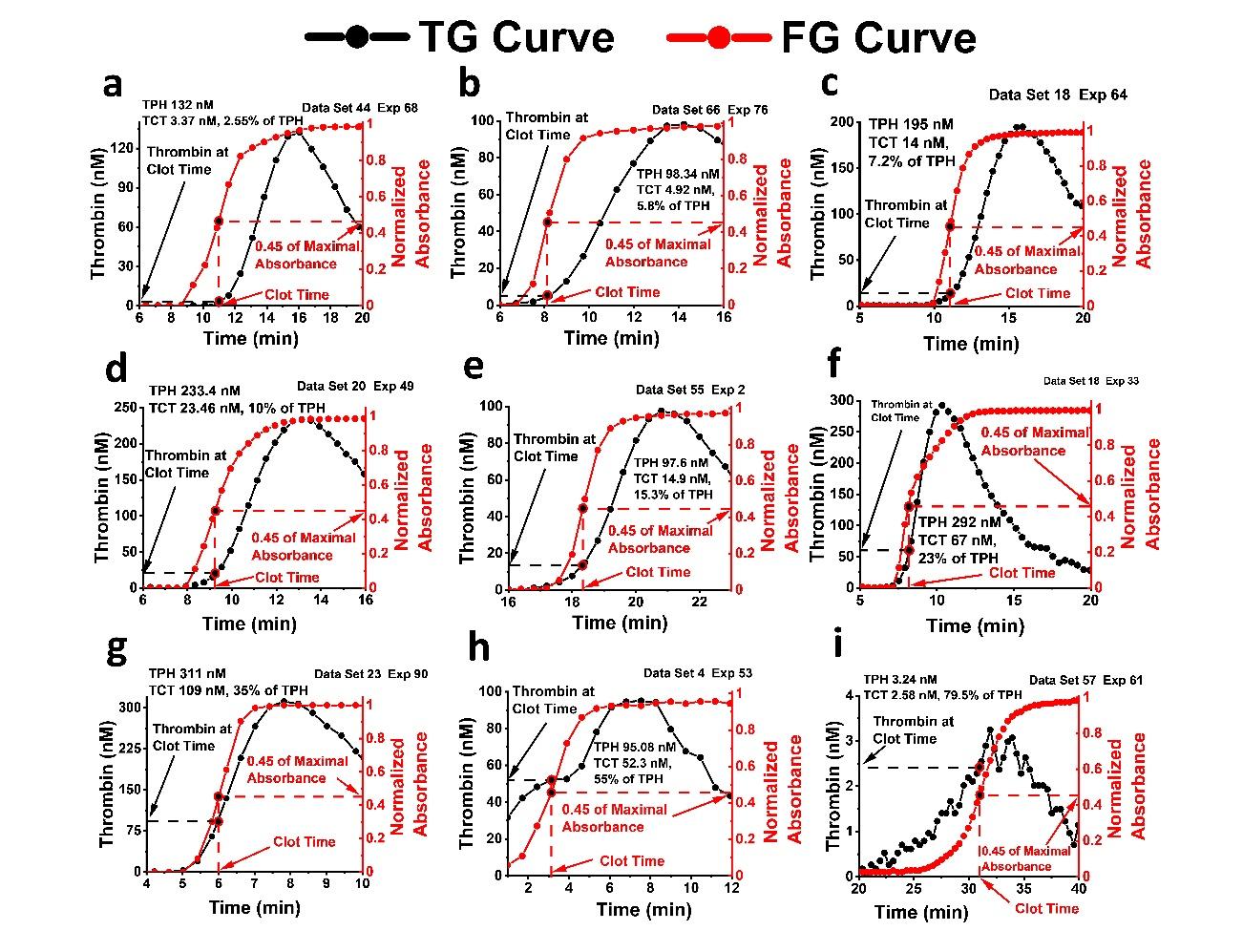


Figure S2. Representative TG and fibrin formation curves with definitions of parameters’ values.


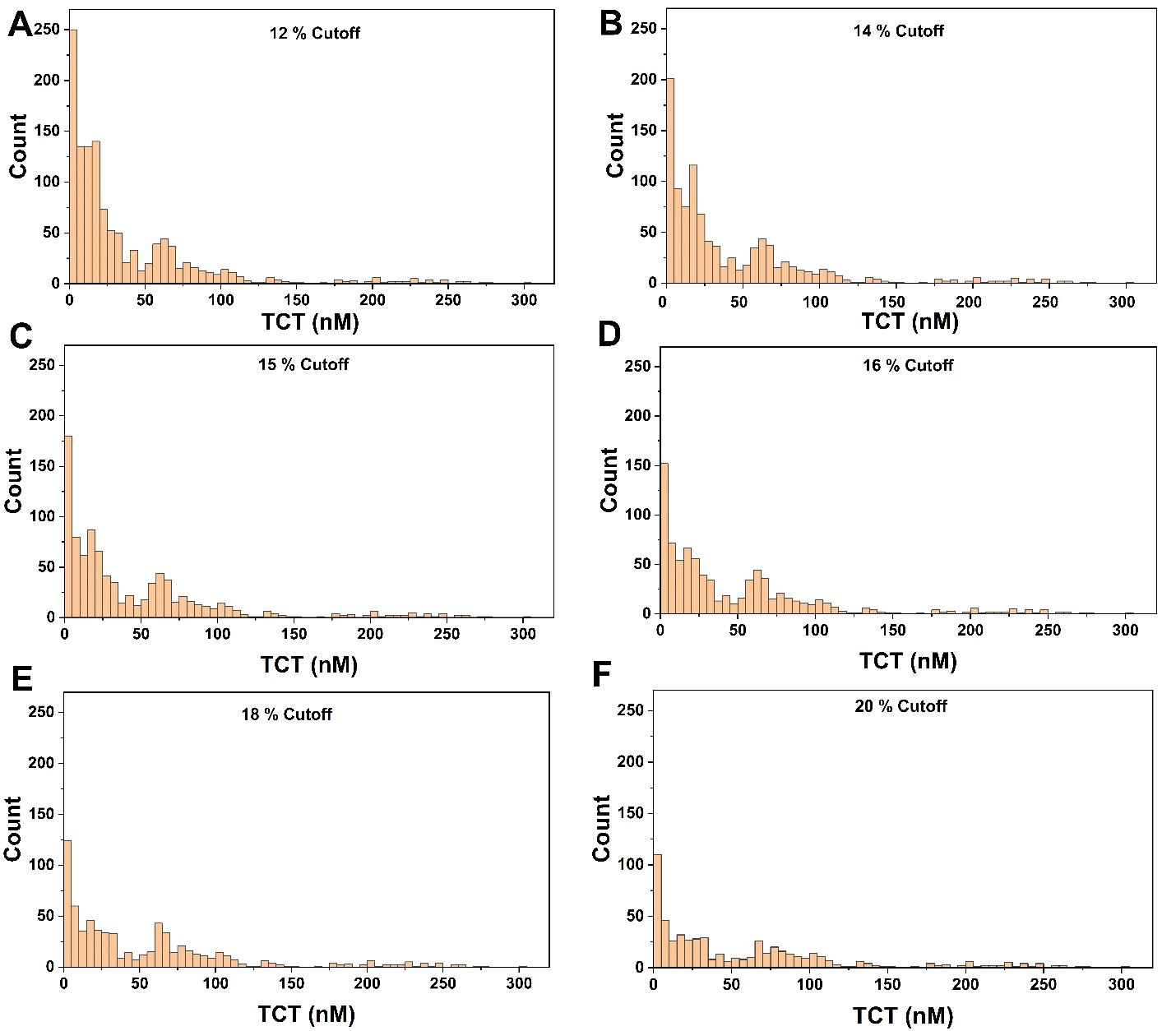


Figure S3. The distributions of subpopulations of experiments with TCT elevating the control cutoff border of 12% (A), 14% (B), 15% (C), 16% (D), 18% (E) and 20% (F).

Table S2. Statistics on the groups of experiments with different PS.

| Procoagulant Score | | <0 | 0 | 1 | 2 | 3 |
| --- | --- | --- | --- | --- | --- | --- |
| Number of cases | | 376 | 2452 | 2456 | 409 | 65 |
| Thrombin Peak Height | Mean (nM) | 47.37 | 81.35 | 119.54 | 155.27 | 305.36 |
|  | 1st quantile (nM) | 10.02 | 33.11 | 52.40 | 86.06 | 154.91 |
|  | Median (nM) | 33.65 | 67.72 | 88.61 | 128.38 | 341.40 |
|  | 3rd quantile (nM) | 67.67 | 109.50 | 163.35 | 174.89 | 369.36 |
| Thrombin production | Mean (nM) | 14.73 | 27.73 | 48.38 | 68.90 | 167.42 |
|  | 1st quantile (nM) | 1.55 | 5.76 | 11.82 | 25.62 | 85.66 |
|  | Median (nM) | 8.59 | 16.23 | 26.03 | 43.35 | 186.51 |
|  | 3rd quantile (nM) | 19.98 | 36.84 | 58.18 | 78.18 | 208.05 |
| Thrombin at Clot Time | Mean (nM) | 7.69 | 7.05 | 12.78 | 21.78 | 61.91 |
|  | 1st quantile (nM) | 1.21 | 1.77 | 3.47 | 6.05 | 43.53 |
|  | Median (nM) | 3.41 | 3.74 | 5.86 | 10.00 | 59.15 |
|  | 3rd quantile (nM) | 7.43 | 9.51 | 12.20 | 20.07 | 86.04 |


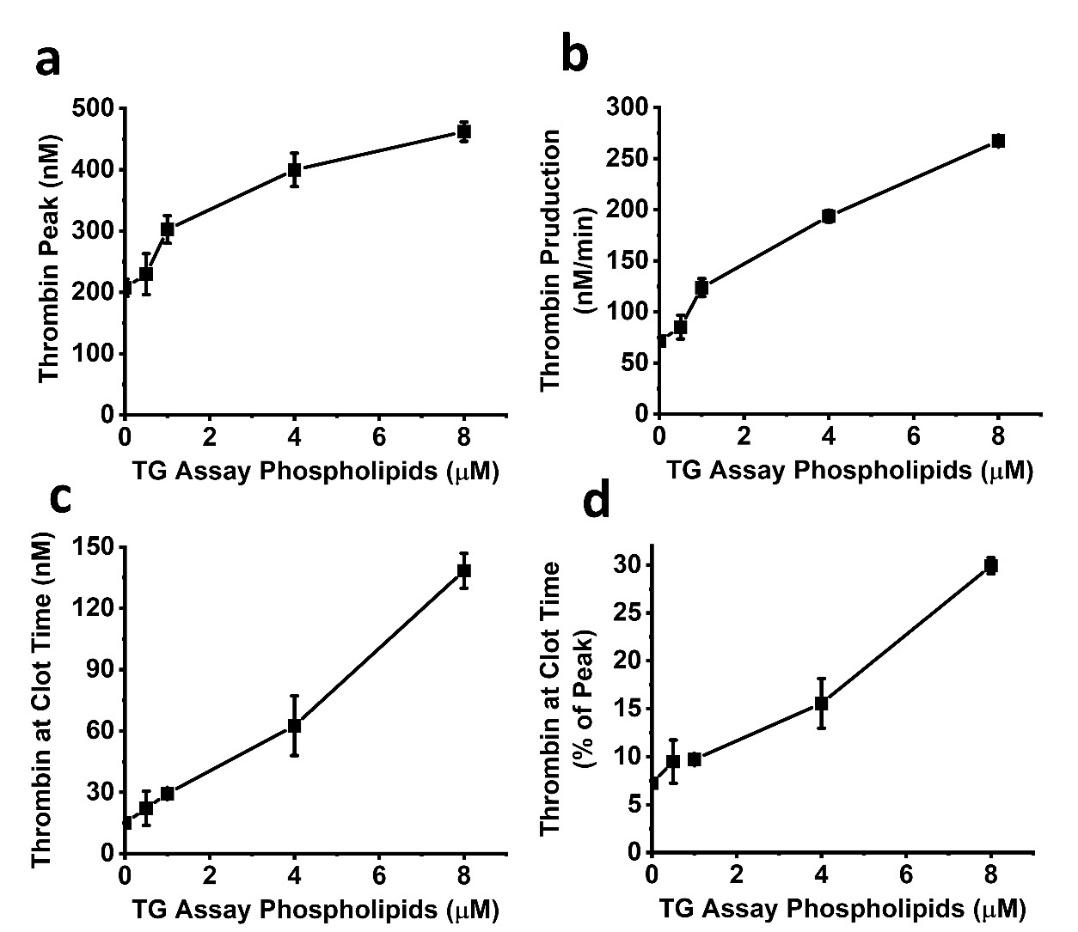


Figure S4. The dependencies of TPH (a), TPR (b) and TCT (c-d) on TG phospholipids concentration in normal plasma. Experiments 73-90 from Dataset 61 from Supplemental Excel Table. Each dot represents a mean value of two duplicate experiments with SD values.


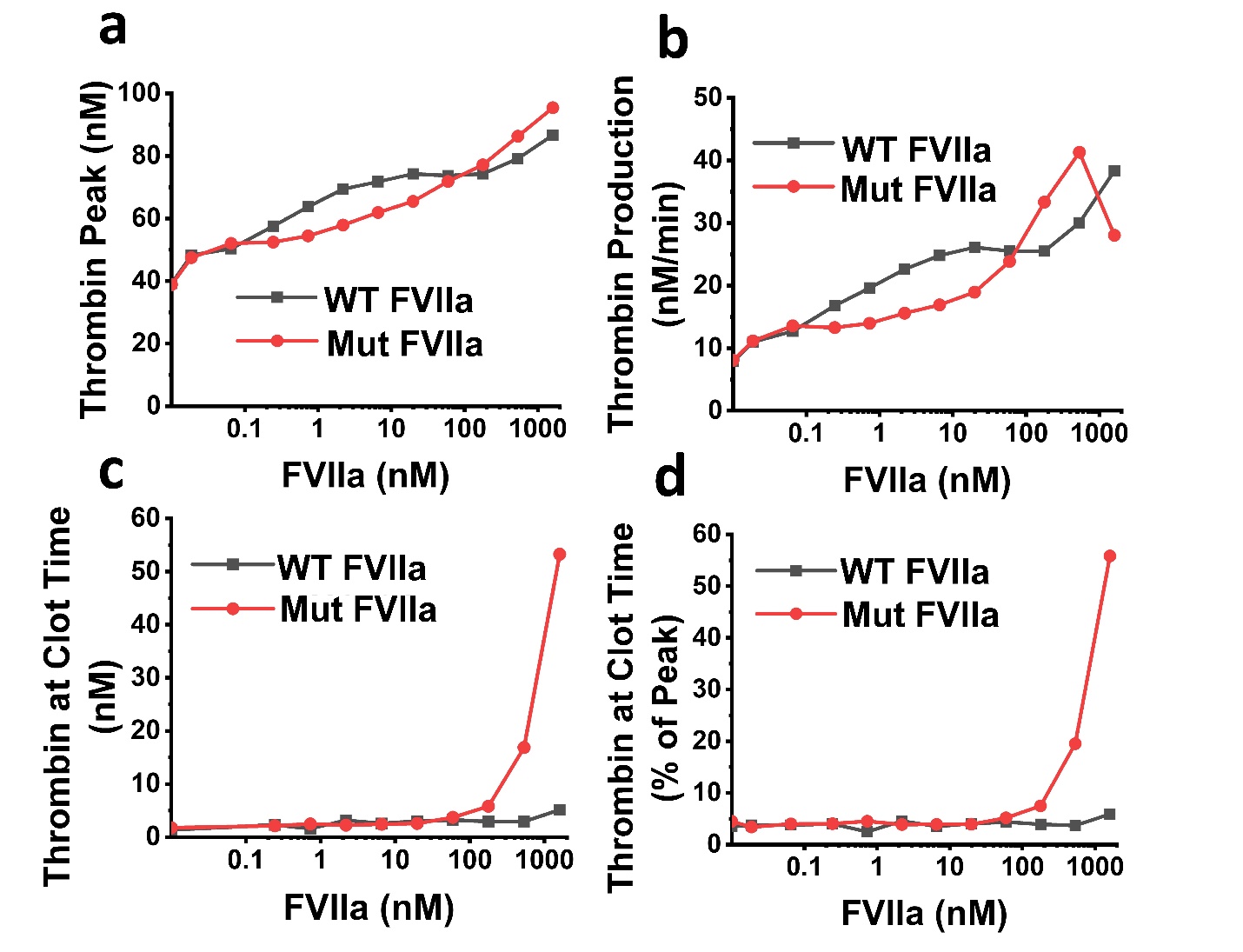


Figure S5. The dependencies of TPH (a), TPR (b), and TCT (c,d) on two types of FVIIa concentration in FVIII-deficient plasma. Dataset 49, experiments 47-71 from Supplemental Excel Table. TF concentration is equal to 0.5 pM. Each dot represents a single TG experiment.


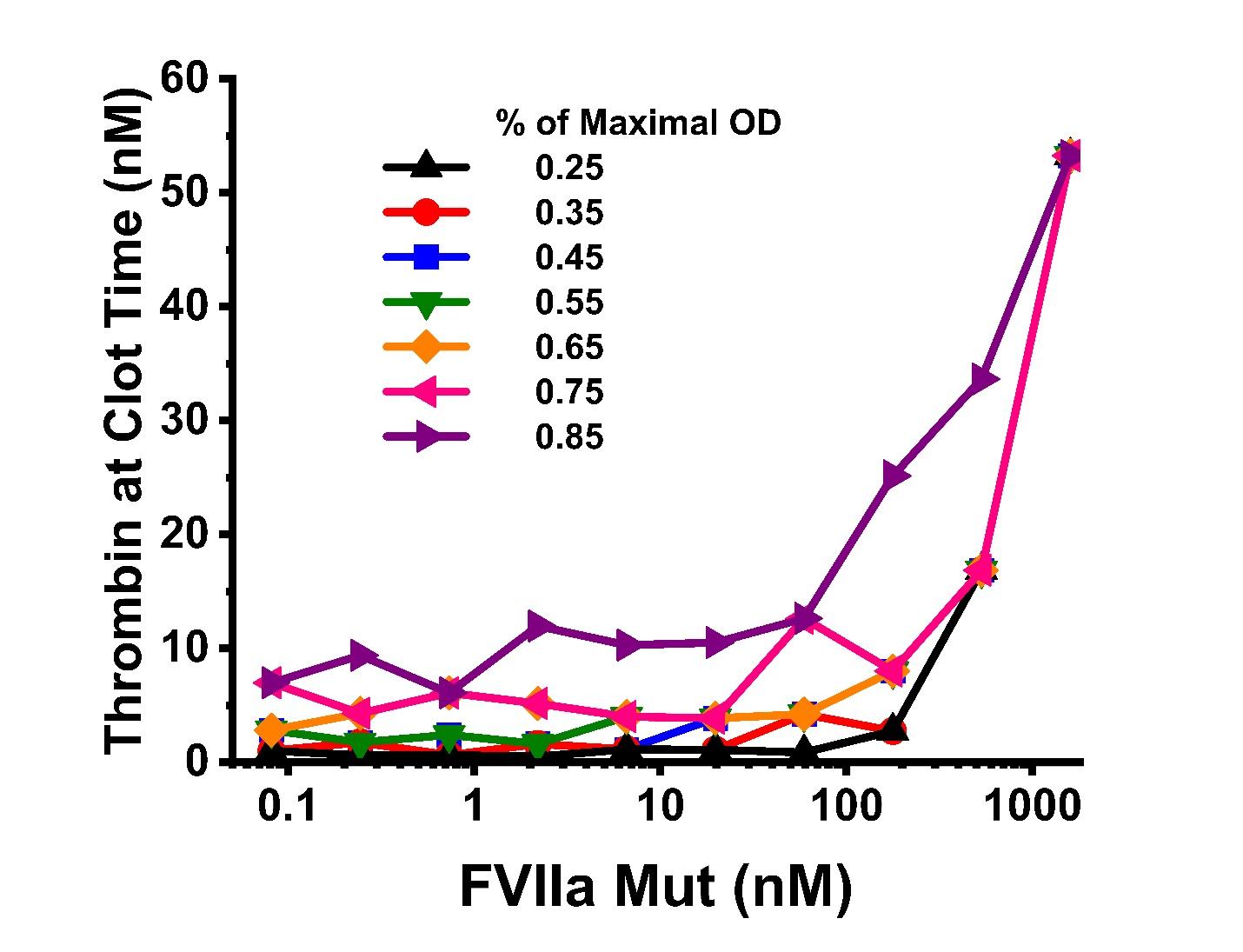


Figure S6. TCT values from experiment in Fig. S5 with different absorbance values chosen for CT calculation. This is an example of how the Thrombin at Clot Time value can change when a cut off changes from 25% to 0.85% of maximal optical density (OD) value. In this example, the Thrombin at Clot Time is shown as a function of FVIIa concentration. Reducing the cut-off from the value used in this study (45% of maximal OD) will shorten the reported clot time values (because optical density reaches the cut-off value sooner), and will therefore reduce the amount of thrombin recorded at the clot time. Conversely, higher cut-off results in longer clot times and higher amounts of thrombin a clot time. However, the shapes of the curves showing the relationship between the Thrombin at Clot Time and FVIIa concentration remained comparable.
